# Exploring the Role of a Novel Peptide from *Allomyrina dichotoma* Larvae in Ameliorating Lipid Metabolism in Obesity

**DOI:** 10.3390/ijms21228537

**Published:** 2020-11-12

**Authors:** Sung Mun Bae, Meiqi Fan, Young-Jin Choi, Yujiao Tang, Gwanghui Jeong, Kyungjae Myung, Byung-gyu Kim, Eun-Kyung Kim

**Affiliations:** 1 Gyeongnam Agricultural Research and Extension Services, Jinju 52733, Korea; smbae@korea.kr (S.M.B.); ghjeong@korea.kr (G.J.); 2 Division of Food Bioscience, College of Biomedical and Health Sciences, Konkuk University, Chungju 27478, Korea; fanmeiqi@kku.ac.kr (M.F.); choijang11@kku.ac.kr (Y.-J.C.); yuanxi00@126.com (Y.T.); 3 School of Bio-Science and Food Engineering, Changchun University of Science and Technology, Changchun 130600, China; 4 Center for Genomic Integrity, Institute for Basic Science, Ulsan 44919, Korea; kjmyung@ibs.re.kr (K.M.); goldenlion@ibs.re.kr (B.-g.K.); 5 Department of Food Science and Nutrition, Dong-A University, Busan 49315, Korea

**Keywords:** *Allomyrina dichotoma* larvae, peptide, lipid metabolism, anti-obesity

## Abstract

The aim of this study was to identify an anti-obesity peptide from *Allomyrina dichotoma* and investigate the lipid metabolic mechanism. Enzymatically hydrolyzed *A. dichotoma* larvae were further separated using tangential flow filtration and consecutive chromatographic processes. Finally, an anti-obesity peptide that showed the highest inhibitory effect on lipid accumulation was obtained, and the sequence was Glu-Ile-Ala-Gln-Asp-Phe-Lys-Thr-Asp-Leu (EIA10). EIA10 decreased lipid aggregation in vitro and significantly reduced the accumulation of body weight gain, liver weight, and adipose tissue weight in high-fat-fed mice. Compared with the control group, the levels of total cholesterol (TC), triglyceride (TG), low-density lipoprotein cholesterol (LDL), insulin, and homeostasis model assessment of insulin resistance (HOMA-IR) in the high-fat diet (HFD) group increased significantly, and the content of high-density lipoprotein cholesterol (HDL) in the serum decreased significantly. On the contrary, the levels of TC, TG, and insulin in the EIA10 group decreased significantly, and the HDL content increased significantly compared with the HFD group. Additionally, EIA10 dramatically decreased mRNA and protein levels of transcription factors involved in lipid adipogenesis. Taken together, our results suggest that EIA10 could be a promising agent for the treatment and prevention of obesity.

## 1. Introduction

The Food and Agricultural Organization of the United Nations (FAO) has recently proposed that edible insects be used, such as the rhinoceros beetle (*Alphitobius diaperinus*), as potential food resources due to their high-quality proteins and vitamins [1,2], which may alleviate food shortage and delay global warming. The Japanese rhinoceros beetle, *Allomyrina dichotoma*, inhabits broad-leaved forests in mountainous habitats and lives for approximately 12 months in the wild [3]. Hence, breeding these beetles would save space, time, and human labor owing to their short generation times and small body sizes [4]. *A. dichotoma* belongs to the family Scarabaeidae and the order Coleoptera. The larvae have been traditionally used to treat liver-related diseases and diabetes [5]. Several studies have demonstrated that a boiled extract of *A. dichotoma* has anti-hepatofibrotic, anti-neoplastic, antibiotic, anti-diabetic, and anti-obesity effects [6,7,8].

Abnormal lipid metabolism refers to the abnormal synthesis, degradation, digestion, and absorption of lipid substances in the body, resulting in excessive lipid in various tissues, which affects various body functions [9]. Abnormal lipid metabolism is mainly caused by excessive energy storage, manifested as cholesterol, triglyceride (TG), and excessive free fatty acid (FFA) levels [10] and causes fatty liver, obesity, diabetes, hyperlipidemia, hypertension, and coronary heart disease [11,12]. Obesity, fat accumulation in abdominal or abdominal viscera, is characterized by the clinical manifestation of abdominal enlargement as well as waist circumference and waist–hip ratio increase. This chronic metabolic disorder is a major global health problem that has been rising rapidly in recent years [13]. Obesity is a major risk factor for systemic inflammation, hyperlipidemia, insulin resistance, and cardiovascular disease [14]. Orlistat, an effective inhibitor of the pancreatic and gastric lipases in the gastrointestinal tract, is now the drug the most widely employed as an anti-obesity medication. However, its usage results in many undesirable intestinal reactions such as fatty feces, diarrhea, and abdominal discomfort [15].

Despite the high prevalence of obesity worldwide, not many new treatments have been approved. Therefore, there is an urgent need for new treatments for obesity. At present, anti-obesity peptide research is a hotspot, with focus on the inhibition of pancreatic lipase activity and appetite suppression. Egg white hydrolysates prepared by proteases and pepsin digestion reduced the lipid content of liver, muscle, and body fat in rats [16,17]. The effects of egg hydrolysates and black soybean hydrolysates on human markers of diabetes and obesity have drawn attention to their absorption and molecular targets [18]. Camel milk peptides possess anti-diabetic and anti-obesity effects [19]. Similarly, fish collagen peptides, derived from water-hydrolyzed components, reduced lipid accumulation and the expression of CCAAT/enhancer-binding protein-*α* (*C/EBP-α*) and peroxisomal proliferator-activated receptor γ (*PPARγ*) genes during the differentiation of 3T3-L1 cells [20]. Oral supplementation with collagen fragments reduces body fat and body weight and improves cytokines [21].

However, to our knowledge, this is the first study investigating the effects of a peptide from *A. dichotoma* larvae, EIAQDFKTDL (Glu-Ile-Ala-Gln-Asp-Phe-Lys-Thr-Asp-Leu, EIA10), on an experimental animal model widely used by pharmaceutical companies to test anti-obesity agents and insulin sensitizers. Our aim was to investigate the improvement in lipid metabolism induced by EIA10 in high-fat diet (HFD)-fed mice characterized by early onset of obesity and several physiological abnormalities of obesity. We hypothesized that ameliorative lipid metabolism due to EIA10 could help to reduce obesity and ameliorate obesity-related metabolic disorders in obese subjects.

## 2. Results and Discussion

### 2.1. Isolation, Purification, and Identification of Novel Peptide, EIA10

**Figure 1:**
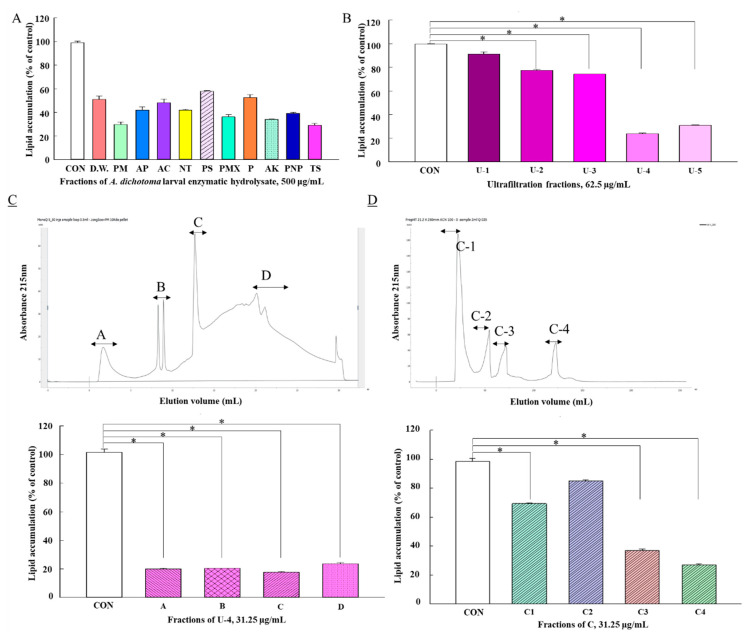
Inhibitory effect of *Allomyrina dichotoma* larvae enzyme hydrolysate on lipid accumulation. (**A**) Inhibition of adipocyte differentiation by enzymatic hydrolysates of *A. dichotoma* larvae. (**B**) Anti-obesity activity of fractions of *A. dichotoma* enzymatic hydrolysate classified by molecular weight. (**C**) Anti-obesity activity (upper panel) and ion-exchange chromatography (lower panel) of 10‒100 kDa ultrafiltration fraction. (**D**) Anti-obesity activity (upper panel) and reversed-phase column fractional chromatogram (lower panel) for preparative ion-exchange chromatography of peak C from (**C**). Lipid accumulation was measured by Oil Red O staining; untreated 3T3-L1 cells were used as control. PM: Promod 278P; AP: Alphalase NP; AC: Vision Biochem, Alcalase; NT: Neutrase; PMX: PS Pepsin Protamex; P: Pancreatin; AK: Food Pro Alkaline protease; PNP: Protease NP; TS: Trypsin. Data are expressed as mean ± standard error of the mean. * *p* < 0.05 vs. control (CON) cells. (*n* = 3 per group).

Recent reports show that food proteins can be converted into biologically active peptides by hydrolysis. In this study, we purified 11 (Figure 1A) enzymatic protein hydrolysates (EPHs) from *A. dichotoma* larvae using ultrafiltration (UF) and ion-exchange chromatography (IEC) to identify the fraction with anti-lipid accumulation activity. The activity was performed by measuring the anti-adipogenic effects of EPHs in 3T3-L1 cells. The optimal concentration was selected by cell viability test (data in Supplementary Materials file). Using UF, five fractions, U-1 (<1 kDa), U-2 (1‒5 kDa), U-3 (5‒10 kDa), U-4 (10‒100 kDa), and U-5 (>100 kDa), were obtained. Among them, U-4 showed the highest anti-adipogenic effect (23.9%) at 62.5 μg/mL of sample concentration and was therefore subjected to IEC (Figure 1B) by which it showed good separation into constituent peaks, i.e., A, B, C, and D, at 215 nm (Figure 1C, upper panel), which were collected and freeze-dried. Peak C showed the highest anti-adipogenic effect (26.9%) at 31.25 μg/mL of sample concentration (Figure 1C, lower panel) and was subsequently separated into C1, C2, C3, and C4. Subsequent separation and identification of anti-obesity peptides from the C-4 fraction were performed by reversed-phase-HPLC and nanoUPLC Orbitrap Fusion Lumos MS (Figure 1D, Table 1). As Glu-Ile-Ala-Gln-Asp-Phe-Lys-Thr-Asp-Leu (EIA10) had 30.22% of lipid accumulation in the 3T3-L1 cells (Table 1), we therefore selected EIA10 for subsequent experiments.

### 2.2. Effects of EIA10 on Body Composition in High-Fat Diet (HFD)-Fed Mice

**Figure 2:**
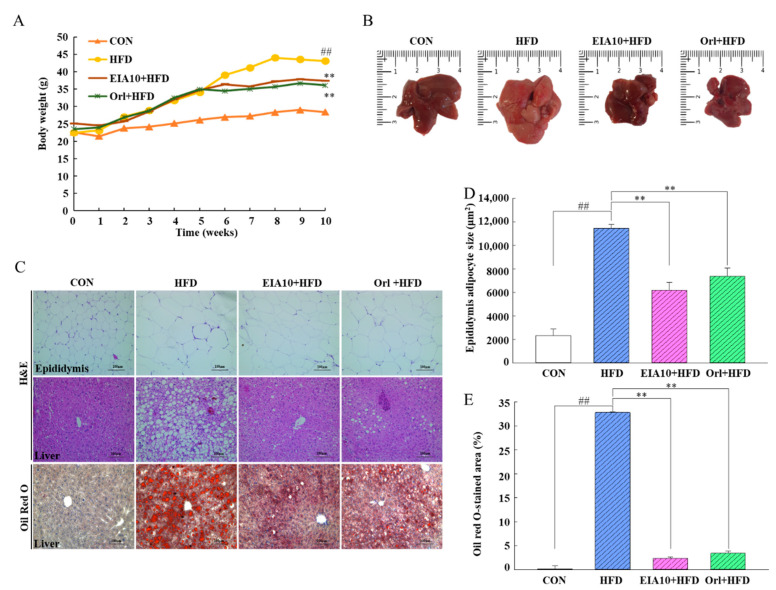
(**A**) Body weight and (**B**) liver histology of HFD group mice. Glu-Ile-Ala-Gln-Asp-Phe-Lys-Thr-Asp-Leu (EIA10) reduces lipid accumulation in the liver and adipose tissue. (**C**) Histological analysis of the liver and epididymal adipose tissue by hematoxylin and eosin (H&E) staining and Oil Red O staining. Scale bar: 100 um (**D**) Mean adipocyte area (μm^2^). (**E**) Mean Oil Red O staining in hepatocytes area (μm^2^). Data are expressed as mean ± standard error of the mean. ^##^ *p* < 0.01 vs. control (CON) group. ** *p* < 0.01 vs. HFD group (*n* = 8 per group). HFD, high-fat diet; Orl, Orlistat.

The impact of EIA10 on body weight and fat composition was evaluated in HFD-fed mice. The experimental design is described in Section 3.8. After five weeks of continuous administration of positive control (orlistat) or EIA10, the weight of HFD-fed mice treated with orlistat and EIA10 decreased significantly compared to that of HFD-fed mice (Figure 2A). The total energy intake over 10 weeks was 1062.21 kcal for the HFD group, 1026.01 kcal for the HFD+EIA10 group, and 1031.35 kcal for the HFD+Orlistat group (Table 2). No significant difference was observed in the food intake between different drug groups, indicating that neither orlistat nor EIA10 had any effect on the appetite of the HFD-fed mice (Table 2). Dual-energy X-ray absorptiometry (DXA) analysis of body composition, fat mass, and body weight revealed that fat in the HFD group appeared as increased liver size and higher amount of white fat tissue (instinctive, subcutaneous, and epididymal) and interscapular darker fat tissue compared to the fat in the control group (Table 2). Interestingly, EIA10 treatment significantly reduced white fat tissue weight and the general dispersion of fat (*p* < 0.05) and completely normalized brown adipose tissue weight (Table 2).

### 2.3. Effects of EIA10 on Lipid Accumulation and Hepatocellular Damage

To confirm the reduction in fat mass after EIA10 treatment, we performed histological analysis of both liver and epididymal fat tissues. The liver was dark red and smooth in the control group, whereas the liver in the HFD group was larger, white, smooth, and greasy on the surface, and had convex edges or medial edges (Figure 2B). The color of the liver in the EIA10 group was healthy. Studies have shown that although the number of fat cells in the body does not increase in adult mice with increase in energy accumulation and weight, the size of the fat cells in the body does increase [22]. After H&E staining, the liver tissue was observed by electron microscopy (Figure 2C) and revealed that control group hepatocytes were arranged in a reasonably orderly manner, with nuclei in the central part of the cells, abundant cytoplasm, and clear cell boundaries. However, in the HFD group, liver cells were loosely arranged, more cells were swollen, and many large fat vacuoles could be seen in the cytoplasm. The nuclei were located at the edges of the cells, and fat droplets were deposited in the cells, indicating severe fatty liver degeneration.

Treatment with EIA10 resulted in a more orderly arrangement of the liver cells, reduced the number of fat vacuoles in the cytoplasm, and altered the shape of the hepatocytes to one similar to that of the control group. Similarly, the epididymal fat tissues of mice stained with H&E showed that the fat cells in the control group were uniform in size and compactly arranged, whereas the fat cells in the HFD group were significantly larger and more disorderly than those in the control group (Figure 2C). The size of the adipose cells in mice in the EIA10 group was lower than that of the HFD group (Figure 2D), suggesting that EIA10 has an inhibitory effect on fat accumulation in adipose tissue induced by feeding with HFD. The results of Oil Red O staining in frozen sections of liver tissue showed that there were large areas of lipid droplets in the livers of HFD group mice, whereas EIA10-treated livers showed a significantly reduced number of lipid droplets in the liver (Figure 2C,E). These results showed that oral administration of EIA10 could effectively reduce the hepatic lipid levels in HFD-fed mice and showed a protective effect on the liver against HFD-induced steatosis.

### 2.4. Effects of EIA10 on Blood Biochemical Parameters

Obesity occurs when energy intake is higher than consumption, and the associated imbalance in lipid synthesis and metabolism of fat results in fatty liver, high blood pressure, diabetes, and other metabolic syndromes [23]. Obesity, accompanied by lipid metabolism disorder, glucose metabolism disorder, and abnormal blood glucose levels, has been shown to lead to increased blood TC, TG, and LDL-C levels [24]. Studies have shown that peptide compounds can potentially improve reverse cholesterol transport, thereby alleviating dyslipidemia in the HFD mouse model and also benefiting obese patients [25]. In this study, HFD was successfully used to induce dyslipidemia in mice, including weight gain, fat accumulation in the liver, and impaired blood glucose tolerance. Compared with the control group, serum levels of TC, TG, and LDL-C were significantly higher in the HFD group (Table 2). EIA10 improved dyslipidemia in HFD-fed obese mice and significantly reduced the levels of TC, TG, and LDL-C in HFD-fed mice (Table 2). Researchers have shown that peptides that can increase serum HDL-C levels and their ability to carry cholesterol [26] and lower LDL-C and TG levels in serum can reduce dyslipidemia [27]. In this study, compared with HFD group mice, EIA10 significantly increased serum HDL-C levels and significantly reduced serum LDL-C and TG levels, thereby inhibiting the accumulation of TG in the liver. Moreover, serum aspartate aminotransferase (AST) and alanine aminotransferase (ALT) levels that were significantly increased in HFD-fed mice were significantly reduced by EIA10 (Table 2). These results indicate that EIA10 can effectively balance lipid levels in HFD mice and alleviate liver injury induced by high-fat diet to some extent.

### 2.5. Effects of EIA10 against Diet-Induced Impaired Glucose Homeostasis and Insulin Resistance

One of the manifestations of obesity is the excessive deposition of TG and the inhibition of fat metabolism by insulin [28]. The resulting influx of large amounts of non-esterified fatty acids (NEFAs) into the liver may further impair liver metabolism, leading to increased liver glucose production and TG-rich lipoprotein levels [29].

**Figure 3:**
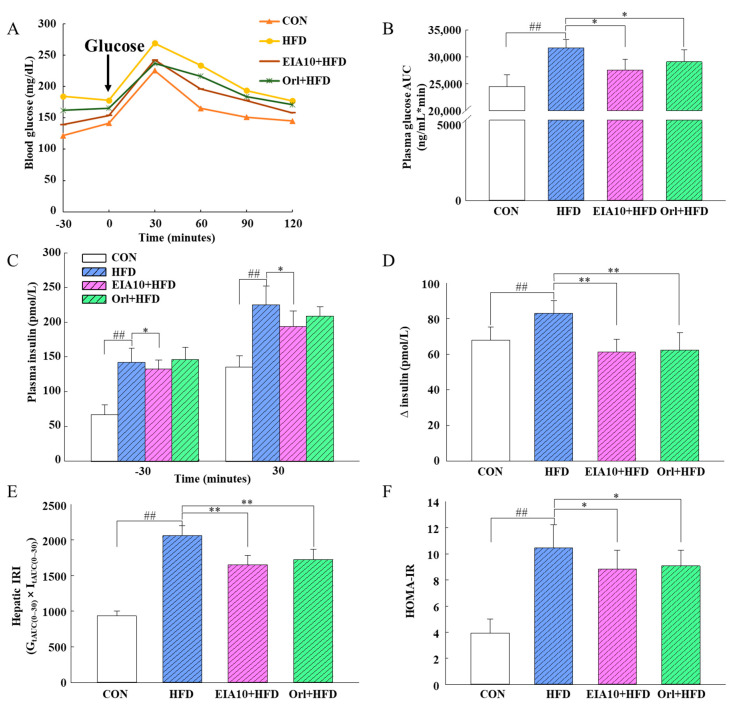
EIA10 treatment improved metabolic parameters of glucose tolerance and insulin resistance in HFD mice. (**A**) Comparison of oral glucose tolerance test results. (**B**) Area under the curve (AUC). (**C**) Plasma insulin concentration (pmol/L) 30 min before (−30) and 30 min after (30) oral glucose administration. (**D**) Glucose-induced insulin secretion after oral glucose administration. (**E**) Hepatic insulin resistance index (hepatic IRI). (**F**) Homeostasis model assessment of insulin resistance (HOMA-IR). Data are expressed as mean ± standard error of the mean. ^##^ *p* < 0.01 vs. control (CON) group. * *p* < 0.05, ** *p* < 0.01 vs. HFD group (*n* = 8 per group). HFD, high-fat diet; Orl, Orlistat.

To compare the differences in glucose metabolism of mice during this experiment, the mice were fasted for 12 h and orally administered glucose at a dose of 1 g/kg, and blood was collected from the tail vein (described in Section 3.12.). Blood glucose concentrations were measured after collection at 30 min, 60 min, 90 min, and 120 min, and serum insulin was measured at −30 min and 30 min. The blood glucose of mice reached a peak 30 min after glucose administration at 250 mg/dL and then declined until it returned to the original level at 120 min (Figure 3A). The oral glucose tolerance test (OGTT) results revealed that, compared with the control group, although the blood glucose level of the HFD group mice still returned to the control group level, the highest value of blood glucose in the HFD group was significantly higher than that of the control group (Figure 3A). 

Mice can be considered diabetic when their fasting blood glucose concentration is >300 mg/dL. In this experiment, none of the mice became diabetic, and their regulation of glucose metabolism remained within normal limits. To further explore the changes in blood glucose concentrations in each group, the area under the OGTT curve (AUC) was calculated (Figure 3B). AUC is derived from the time–glucose concentration curve, which reflects the geometric mean of the five-point blood glucose levels in the OGTT. Compared with the blood glucose at single points, the AUC is a better indicator of the changing trend in blood glucose levels over time. The area under the curve (AUC) in mice showed that the AUC values of the EIA10 and orlistat groups were significantly lower than those of the HFD group (*p* < 0.05, Figure 3B). Insulin resistance (IR) is the central link and pathogenesis of various metabolic abnormalities [30]. IR also increases the content of FFAs and TGs, reduces the inhibitory effect of insulin on gluconeogenesis and glucose release, and leads to the increase of blood glucose [31]. In this study, to further investigate the impact of EIA10 on glucose and lipid metabolism, an insulin resistance experiment was conducted. The results showed that, compared with the CON group, the insulin secretion level of HFD mice was significantly increased after oral glucose. Compared with HFD, the insulin secretion level of the EIA10 group decreased after oral glucose, and the difference was significant (*p* < 0.05, Figure 3C,D).

In this study, the mice in the HFD group showed increased TC, TG, LDL, hepatic insulin resistance index (IRI), and HDL levels (Table 2, Figure 3E,F), which was consistent with the impairment of glucose and lipid metabolism at the onset of obesity. The mice in the HFD group showed liver steatosis, which is closely related to obesity. Five weeks of oral EIA10 intervention improved glucose and lipid metabolism and alleviated IR in the obese mice to some extent. 

Orlistat is currently the only non-prescription drug available for weight loss and was used as a positive control in this study. Compared with orlistat, EIA10 has all natural, non-toxic benefits. The results of this experiment showed that both orlistat and EIA10 could inhibit lipid accumulation and significantly reduce TC and TG levels in serum in terms of reducing body fat content, and there was no significant difference between them. There are many intolerable side effects of orlistat including diarrhea, flatulence, and fecal incontinence. However, EIA10 is assumed to have a benign regulatory effect on the development of obesity and lipid metabolism as an all-natural agent. Therefore, it is in need of further investigation as to whether EIA10 causes side effects such as fecal incontinence and diarrhea, and which organs are targeted by EIA10 in a clinical study. 

### 2.6. Effects of EIA10 on Lipogenic Molecule Signaling Pathway

**Figure 4:**
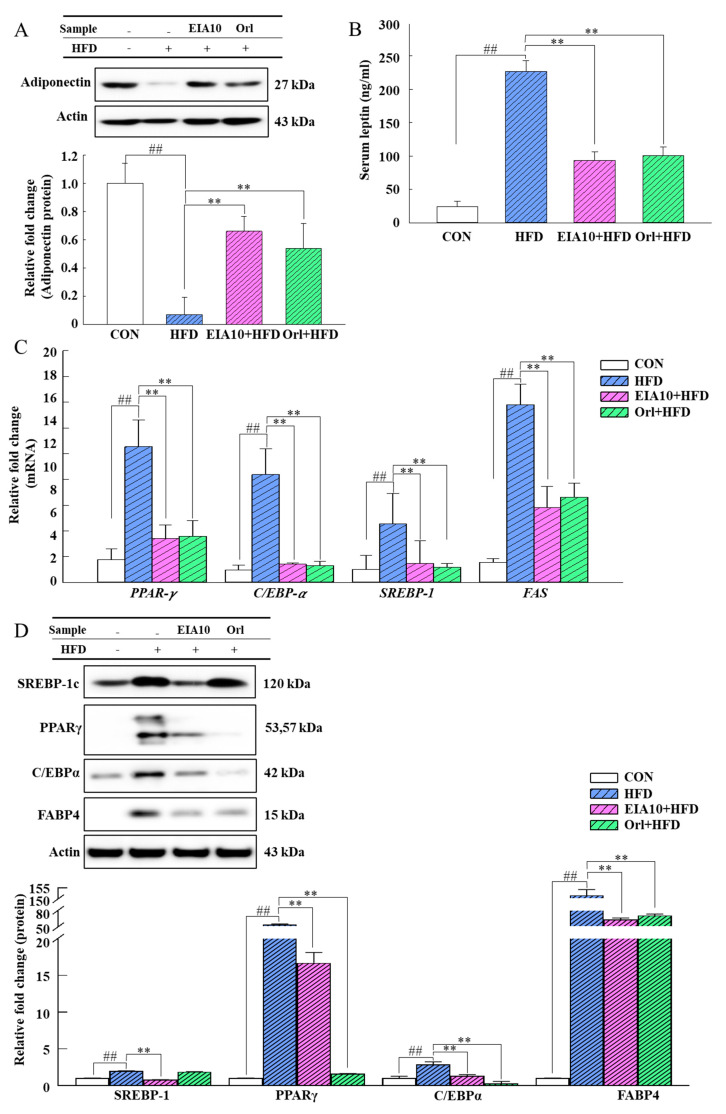
Effect of EIA10 on protein levels of (**A**) adiponectin in mouse epididymal adipose tissue and (**B**) serum leptin. (**C**) mRNA levels of transcription factors in mouse epididymal adipose tissue quantified by real-time PCR. (**D**) Western blotting. “+” and “–” meaning with or without HFD treatment. Data are expressed as mean ± standard error of the mean. ^##^ *p* < 0.01 vs. control (CON) group. ** *p* < 0.01 vs. HFD group (*n* = 8 per group). HFD, high-fat diet; Orl, Orlistat.

Adiponectin activates AMPK in adipose tissue and inhibits sterol regulatory element-binding transcription factor 1 (*SREBP-1c*) precursor cleavage and nuclear translocation, resulting in the inhibition of *SREBP-1c*-mediated adipogenesis [32]. In this study, we measured the protein levels of adiponectin in mouse epididymal adipose tissue and observed that EIA10 improved the adiponectin. Therefore, EIA10 could inhibit fat accumulation in mice by improving the adiponectin and reducing the plasma leptin concentration, thereby reducing fat synthesis (Figure 4A,B). SREBPs are closely related to a series of metabolic diseases including dyslipidemia, insulin resistance, diabetes, and nonalcoholic fatty liver disease. Lipid-lowering effects caused by several other peptide compounds have also been attributed to the decrease in *SREBP-1c* [33]. In this experiment, mRNA and protein levels of *SREBP-1c* were lower in the adipose tissue of HFD-fed mice treated with EIA10 than in the HFD group (Figure 4C,D), suggesting that EIA10 regulates fatty acid synthesis by regulating the expression of *SREBP-1c* to lower lipid levels.

**Figure 5:**
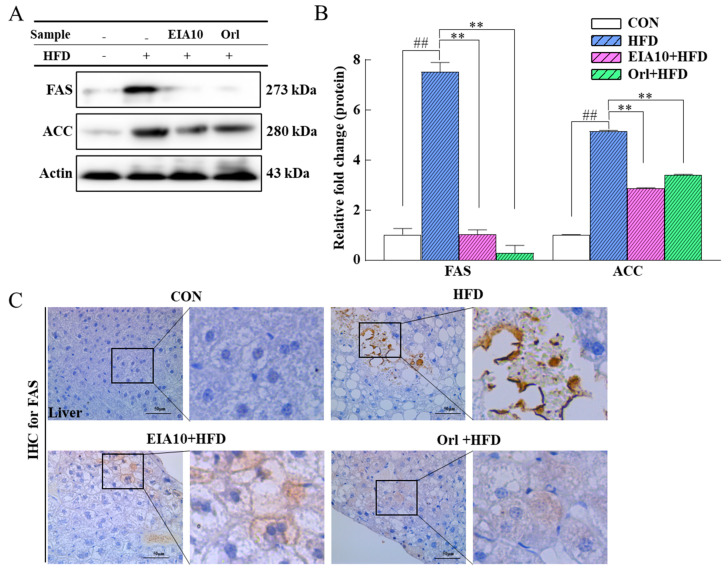
Effect of EIA10 on (**A**,**B**) protein of lipogenic enzyme in epididymal adipose tissue of animals treated with different groups as quantified by Western blot. (“+” and “–” meaning with or without HFD treatment). (**C**) IHC-stained *FAS* is shown in mouse liver tissues (left and right images in C, right images in C represent areas in the black rectangles). Scale bar: 50um Data are expressed as mean ± standard error of the mean. ^##^ *p* < 0.01 vs. control (CON) group. ** *p* < 0.01 vs. HFD group (*n* = 8 per group). HFD, high-fat diet; Orl, Orlistat.

To further clarify the lipid-lowering mechanism of EIA10, we investigated the inhibitory effects of EIA10 on fatty acid synthase (*FAS*) and acetyl-CoA carboxylase (*ACC*) gene expression level. *FAS* is a polypeptide chain with distinct enzyme activity necessary for fatty acid biosynthesis. In the cytoplasm, acetyl-CoA is carboxylated by acetyl-CoA carboxylase (*ACC*) to form malonyl-CoA. Malonyl-CoA and nicotinamide adenine dinucleotide phosphate (NADPH) are used by *FAS* to produce palmitic acid, which is the most common saturated fat in plants and animals [34,35]. Therefore, *FAS* inhibition becomes an essential goal for weight prevention and lipid modulation. The results of this experiment showed that EIA10 had an inhibitory effect on both *FAS* and *ACC* gene expression (Figure 5A,B), indicating that EIA10 could effectively inhibit the synthesis and accumulation of fat in vivo, which may be one of the mechanisms of EIA10 to improve obesity and lipid metabolism in mice. We observed the effect of EIA10 on *FAS* activity in liver tissues by immunohistochemistry (IHC). The results showed that EIA10 also inhibited *FAS* in liver tissue (Figure 5C).

*FAS* is regulated by peroxisomal proliferator-activated receptor γ (*PPARγ*), and its upregulation increases the amount of lipid synthesized from acetyl-CoA, causing the accumulation of TGs in liver tissues. In this study, we found that EIA10 reduced HFD-induced fat synthesis and accumulation through *SREBP-1c* regulation, leading to lipid reduction. Most of the genes involved in adipogenesis are induced and regulated by *PPARγ* and *C/EBP-α* [36]. As shown in Figure 4C,D, EIA10 decreased the mRNA and protein levels of *PPARγ* in the HFD group mice. *FABP4* is a target gene of *PPARγ* and is involved in adipocyte differentiation [37]. In this study, we found that EIA10 downregulated the expression of *PPARγ*, *C/EBP-α*, *FAS*, and *SREBP-1c*, and also significantly decreased the protein levels of *PPARγ*, *C/EBP-α*, *SREBP-1c*, and *FABP4* (Figure 4C,D).

## 3. Materials and Methods

### 3.1. Biological Materials

The third instar *A. dichotoma* larvae used in the experiment were purchased from Geoje Insect Farm (Gyeongnam-si, Gyeongsangbuk-do, Korea) in 2019. The larvae were maintained on a diet of fermented sawdust, and third instar larvae were collected based on the size of their head capsules. We used third instar larvae for this study because they have a longer larvae period and eat a large amount of feed compared to first or second instar larvae. The insects were purchased and used after washing and freezing. Pepsin and trypsin were obtained from Sigma-Aldrich (St Louis, MO, USA); Protease NP (PNP), Pancreatin (*p*), Alphalase NP (AP), Food Pro Alkaline protease (AK), Promod 278P (PM) Vision Biochem, Alcalase (AC), Neutrase (NT), and Protamex (PMX) were purchased from Daejong Corporation.

### 3.2. Enzymatic Hydrolysis

*A. dichotoma* larvae were lyophilized (Lyoph-Pride 100, Ilshinbiobae, Dongducheon-si, Korea), pulverized into a powder using a grinder (VMO159A, Vitamix, Cleveland, OH, USA), and hydrolyzed with enzymes according to the method described by Park et al. [38]. Table 3 summarizes the optimal pH, temperature, and characterization of different enzymes. Briefly, 100 mL of distilled water were added to 10 mg of the freeze-dried *A. dichotoma* larvae powder followed by 0.1 μL (or mg) of an enzyme. The enzymatic hydrolysis was performed for 6 h to achieve an optimal hydrolytic level and was followed by direct heating at 100 °C for 10 min to inactivate the enzymes. After the enzyme-inactivated mixture was centrifuged at 2000 rpm (Optima XE100 ultracentrifuge, Beckman Coulter, Miami, FL, USA), the supernatant was vacuum-filtered and subjected to ultrafiltration (UF, Ultracel PL-10, Millipore, Billerica, MA, USA) with Amicon^®^ Stirred Cells (Millipore, Billerica, MA, USA) membranes (membrane cut-off: 100 kDa, 10 kDa, 5 kDa, 1 kDa) in a nitrogen atmosphere at a pressure of 5 bar. The proteins were fractionated from 100 kDa or higher to 10 kDa, 10 kDa to 5 kDa, 5 kDa to 1 kDa, or 1 kDa or lower molecular weight, filtered, and lyophilized to obtain the hydrolysate, which was stored at −20 °C until further use.

### 3.3. Cell Culture, Differentiation, and Treatments

The 3T3-L1 cells were obtained from the Korean Cell Line Bank (Seoul, Korea). They were cultured and maintained in Dulbecco’s modified Eagle medium (DMEM) at 37 °C in a humidified atmosphere of 5% CO_2_. Additionally, 2-day post-confluent preadipocytes (designated day 0) were cultured in a 2-day differentiation medium composed of DMEM, 0.5 mM IBMX, 1 μM dexamethasone, and 5 μg/mL insulin to induce differentiation. The cells were then cultured in a DMEM containing 5 μg/mL insulin for another 2 days. The medium was replaced every 2 days until the cells had accumulated intracellular lipid droplets [39].

### 3.4. Determination of the Deposition of Lipids by Oil Red O Staining

Cells were seeded in 6-well plates, and adipocyte differentiation was induced for 8 days. On day 8, the cells were fixed with 10% buffered formalin and stained with Oil Red O solution for 60 min at room temperature. Images of lipid droplets in the 3T3-L1 adipocytes were captured using a Leica DMi1 microscope (Leica, Germany).

### 3.5. Fast Protein liquid Chromatography (FPLC)

To separate and purify the fraction possessing anti-obesity efficacy obtained by UF into a single-peptide fragment, the ultrafiltrate was separated and purified step by step using three FPLC columns (Akta Avant 25, GE Healthcare, Uppsala, Sweden). Ion-exchange chromatography (IEC) was performed using Mono-Q 5/50 GL (5*50 mm, 10 µm), using binding buffer (50 mM phosphate buffer (pH 7)) and elution buffer (1 M NaCl‒phosphate buffer, pH 7). The elution buffer concentration was increased from 0% to 100%, the column volume value was 20, flow rate 2 mL/min, and absorbance 280 nm. After IEC, HiPrep 26/10 desalting column binding buffer (50 mM phosphate buffer (pH 7)) was used at a flow rate of 10 mL/min to remove the salt in the sample. Next, reversed-phase, preparative fractionation was used to separate and purify the fractions with anti-obesity efficacy from the IEC eluate. Separation and purification were performed using an Agilent Zorbax 300SB-C18 PrepHT (7 μm, 21.2 × 250 mm) column with acetonitrile (ACN) (gradient from 100% to 0%) and water as the mobile phase at a flow rate of 3 mL/min and absorbance of 280 nm. The active phase fraction from the reversed-phase preparative analysis was analyzed at 280 nm at a flow rate of 0.5 mL/min at 45% ACN with an Agilent Zorbax 300SB-C18 (5 μm, 4.6 × 250 mm) reversed-phase column.

### 3.6. Analysis of Target Peptides

For nanoUPLC Orbitrap Fusion Lumos MS (Thermo Scientific^TM^, Waltham, MA, USA), peptide digests were done using online reversed-phase chromatography. Peptides above the threshold of 5e3 were selected for fragmentation with dynamic exclusion after a 15-s scan at 10 ppm tolerance. Spectra were searched against the UniProt-*A. dichotoma* database using Proteome Discoverer Sorcerer 2.2.2 with an SEquest-based search algorithm. Comparative analysis of proteins identified in this study was performed using Scaffold 4 Q+S. Protein probabilities were assigned by the Peptide Prophet algorithm [40] with Scaffolds delta-mass correction. Protein identifications were accepted if they could be established at > 99.0% probability to achieve an FDR < 0.1%. Protein probabilities were assigned by the Protein Prophet algorithm [41]. Peptide identifications and protein probabilities were accepted at > 11.0%, and at least 2 identified peptides were grouped based on similar peptides that could not be differentiated based on parsimony/parsimony principles. For peptide and protein comparative analysis, MS/MS analysis alone was used to verify the peptide identifications. For protein analysis, comparative analysis of the peptides and proteins was done using MS/MS analysis alone using MSMS and MSMS analysis with a scaffolding algorithm, and the protein probability was assigned to tandem (sample Mudpit_A4_2: A4_1). The data were deposited into the ProteomeXchange Consortium via the PRIDE [42] partner repository with the dataset identifier PXD019661 and 10.6019/PXD019661.

### 3.7. Peptide Synthesis

The peptides listed in Table 1 were provided by Sewon Biotech (Seoul, Korea), and the method for preparing the peptides is briefly described below. Peptides were synthesized by solid-phase peptide synthesis using fluorenylmethylocycarbonyl-(Fmoc)/tertiary-butyl-(tBu) protocols, and the assembly of amino acid residues was performed in the Syro-I automatic peptide synthesizer (Biotage, Sweden). 2-Chlorotrityl resin (Beadtech, Korea) was used during synthesis and for all first-amino acid attachments. Introduction of Fmoc-amino acids was carried out with a mixture of hexafluorophosphate benzotriazole tetramethyl uronium/1-hydroxybenzotriazole/diisopropylethylamine (1:1:2), and Fmoc groups were removed by treating the resin with 20% piperidine in dimethylformamide.

After assembling all amino acid residues, the crude peptides were cleaved from the peptide resin with a mixture of trifluoroacetic acid/triisopropylsilane/water (95:2.5:2.5) for 2 h at room temperature. Peptide precipitations were then isolated from the cleavage solution with cold diethyl ether and washed four times with cold ether. The purity and the identity of peptides were confirmed by analytical high-performance liquid chromatography (HPLC) (Agilent 1100, Agilent Technologies, Santa Clara, CA, USA) and by liquid chromatography–mass spectrometry (LC/MS) (Thermo Scientific™ LTQ XLTM IonTrap mass spectrometer, San Jose, CA, USA). Following the characterization of the products, the peptides were purified by preparative HPLC (Waters 2998, Milford, MA, USA). The purified peptides were lyophilized, and homogeneity (≥95%) of the compound was confirmed by analytical HPLC and by LC/MS. Finally, each peptide was sequenced using de novo sequencing with the Xcalibar Qual Browser program (ver. 4.2.47, Thermo Fisher Scientific, Bremen, Germany).

### 3.8. Experimental Animals, Diet, and Treatments

Four-week-old male C57BL/6 mice were purchased from the Nara Bio Animal Center (NARA Biotech, Seoul, Korea) All experiments were approved by the Institutional Animal Care and Use Committee of Konkuk University (14 July 2019). All possible efforts were made to minimize the suffering and the animal count utilized in this study (KU19102). The mice were randomly divided into the following two groups after 1 week of acclimatization: *n* = 8 in the control group, fed a normal diet (Control, fat content of 10% kcal), and *n* = 30, fed a high-fat diet (HFD, fat content of 60% kcal). Mice were registered daily for food intake, and their body weights were assessed twice a week. After 5 weeks, mice with a body weight 20% higher than that of the control group were picked and divided randomly into three groups (*n* = 8/group): HFD + EIA10, 100 mg/kg/d; and HFD + orlistat, 60 mg/kg /d for 5 weeks. As a positive control, Orlistat was used. After 12 weeks, the mice were subjected to fasting overnight and sacrificed (Figure 2A). Blood samples were harvested for further analysis. Sections of epididymal adipose and liver tissues were fixed in 10% formaldehyde for histological analyses and frozen in liquid nitrogen.

### 3.9. Body Fat Composition Analysis

Dual-energy X-ray absorptiometry (DXA) measurements were used to compare the body fat. DXA measurements were performed after 4 weeks of administration using a total-body scanner (InAlyzer dual X-ray absorptiometry, Medikors, Gyeonggi, Korea). Data were obtained once with low energy and once with high energy.

### 3.10. Histological Analysis

Liver and epididymal adipose tissues were dissected and embedded in paraffin for histological examination. Tissue blocks were cut into 4-μm thick sections that were stained with hematoxylin and eosin. Frozen liver sections were cut into 8-μm thick sections that were stained with Oil Red O. Adipocyte sizes were assessed using a microscope under 200× magnification to measure tissue size.

### 3.11. Biochemical Analysis

Through heart puncture, blood samples were collected and serum was isolated through centrifugation (3000 rpm for 20 min) and processed before further study at −80 °C. An automated analyzer (Abaxis VETSVAN VS2 Chemistry Analyzer, Union City, CA, USA) assessed serum levels of blood urea nitrogen, alanine aminotransferase (ALT), alkaline phosphatase, aspartate aminotransferase (AST), total protein, serum albumin, and electrolytes. Using a fast blood lipid analyzer (OSANG Healthcare Lipid Pro, Anyang, Korea), total cholesterol (TC), low-density lipoprotein cholesterol (LDL), high-density lipoprotein cholesterol (HDL), and TG were calculated. Adiponectin was analyzed by Western blotting, and leptin was quantified using a leptin enzyme-linked immunosorbent assay (ELISA) kit (MERCK, DHB, Germany).

### 3.12. Oral Glucose Tolerance Test (OGTT) and Insulin Levels

An oral glucose tolerance test (OGTT) to determine the clearance of a standardized glucose load was performed after 5 weeks of oral administration. Plasma glucose was determined under postabsorptive conditions with blood collected at time ‒30 and 0 and then after oral administration of 1 g/kg of glucose at 30, 60, 90, and 120 min. Using a Blood Glucose Testing System (891 Hogye-Dong Dongan-gu, Anyang, Gyeonggi-do, Korea), blood samples were measured from the tail veins. A mouse ultrasensitive insulin ELISA package (ALPCO, Salem, NH, USA) was quantified for serum insulin.

### 3.13. Estimation of Insulin Resistance Indices

The hepatic insulin resistance index (hepatic IRI) was estimated by using the validated method of Matsuda and DeFronzo [43]. Insulin resistance was assessed using the homeostasis model assessment of insulin resistance (HOMA-IR). HOMA is a mathematically derived model of glucose–insulin feedback [44].

### 3.14. mRNA Analysis

Total cellular RNA was isolated from the epididymal adipose tissue of each group of mice using TRIzol. The first strand of complementary DNA (cDNA) was synthesized using Superscript II reverse transcriptase (Invitrogen). The conditions for RT-PCR were similar to those previously described [45]. Quantitative real-time PCR was carried out using a Thermal Cycler Dice TP850 (Takarabio Inc., Shiga, Japan) according to the manufacturer’s protocol. The primers used for gene expression analysis are presented in Table 4.

### 3.15. Protein Quantification

Frozen epididymal adipose tissues from mice were homogenized in liquid nitrogen. Tissue lyses were lysed in a radioimmunoprecipitation lysis buffer containing 1% protein inhibitor cocktail [46]. They were transferred to a nitrocellulose blotting membrane and blocked with 5% skim milk, incubated overnight with primary antibodies purchased from Cell Signaling Technology, and then incubated with secondary antibodies for 2 h. Equal protein loading was ascertained using Ponceau S staining of the blotted membranes. With the help of an improved chemiluminescence detection reagent, the antibody-specific proteins were visualized and quantified using ImageJ tools.

### 3.16. Statistical Analysis

The results shown in this study are from at least three experiments. The data are expressed as the mean ± standard error of the mean. The data were analyzed using SPSS version 11.5 for Windows (SPSS Inc., Chicago, IL, USA). The one-way variance analysis (ANOVA) research of post-hoc analyses by the Student–Newman–Keuls test carried out multi-group analyses of the means. To equate the means of two or more sets of variables that were not usually distributed, Dunnett’s multiple range test was used. Statistically significant was set at *p* < 0.05 and *p* < 0.01.

## 4. Conclusions

This study describes the regulation of lipid metabolism by novel peptide EIA10 from *A. dichotoma* larvae, which helps to extend the nutritive and medicinal value of *A. dichotoma* larvae. *A. dichotoma* larvae were enzymatically hydrolyzed and further purified using tangential flow filtration and different chromatographic methods to obtain Gln-Ile-Ala-Gln-Asp-Phe-Lys-Thr-Asp-Leu (EIA10) as the peptide with the highest anti-adipogenic effect among all of the larvae peptides. EIA10 showed the lowest lipid accumulation of 3T3-L1 adipocyte lipid droplets in vitro.

EIA10 effectively alleviated the symptoms of fatty liver, reduced lipid accumulation, catalyzed the decomposition of TG and TC, and reduced blood lipid levels and body weight of HFD-fed mice. Lipid synthesis can be prevented by inhibiting the expression of related genes. EIA10 inhibited *PPARγ* signaling as well as the development and progression of obesity. It can be seen that clinical treatment using EIA10 for lipid regulation and weight loss has a good development prospect.

## Figures and Tables

**Table 1:** Peptides isolated from *Allomyrina dichotoma* larvae.

No.	Peptide Sequence	Peptide Length	Molecular Composition	Molecular Weight (Da)	Lipid Accumulation (% of Control)
1	KLPFQR (Lys-Leu-Pro-Phe-Gln-Arg)	6	C37 H61 N11 O8	787	57.85
2	STELLIR (Ser-Thr-Glu-Leu-Leu-Ile-Arg)	7	C36 H66 N10 O12	830	59.04
3	EIAQDFK (Glu-Ile-Ala-Gln-Asp-Phe-Lys)	7	C38 H59 N9 O13	849	52.20
4	HLQLAIR (His-Leu-Gln-Leu-Ala-Ile-Arg)	7	C38 H67 N13 O9	849	63.26
5	AVQGLLK (Ala-Val-Gln-Gly-Leu-Leu-Lys)	7	C33 H61 N9 O9	727	48.40
6	IAQGGVLP (Ile-Ala-Gln-Gly-Gly-Val-Leu-Pro)	8	C34 H59 N9 O10	753	38.01
7	NDEELNKLL (Asn-Asp-Glu-Glu-Leu-Asn-Lys-Leu-Leu)	9	C46 H78 N12 O18	1086	39.44
8	AGLQFPVGR (Ala-Gly-Leu-Gln-Phe-Pro-Val-Gly-Arg)	9	C43 H69 N13 O11	943	33.70
9	YRPGTVALR (Tyr-Arg-Pro-Gly-Thr-Val-Ala-Leu-Arg)	9	C46 H77 N15 O12	1031	40.63
10	KSTGGKAPR (Lys-Ser-Thr-Gly-Gly-Lys-Ala-Pro-Arg)	9	C37 H68 N14 O12	900	48.40
11	KQLATKAAR (Lys-Gln-Leu-Ala-Thr-Lys-Ala-Ala-Arg)	9	C42 H79 N15 O12	985	94.26
12	RFQSSAVMA (Arg-Phe-Gln-Ser-Ser-Ala-Val-Met-Ala)	9	C42 H69 N13 O13 S1	995	57.47
13	TLSDYNIQK (Thr-Leu-Ser-Asp-Tyr-Asn-Ile-Gln-Lys)	9	C47 H76 N12 O17	1080	68.00
14	NKLLSGVTIA (Asn-Lys-Leu-Leu-Ser-Gly-Val-Thr-Ile-Ala)	10	C45 H82 N12 O14	1014	77.76
* 15	**EIAQDFKTDL** **(Glu-Ile-Ala-Gln-Asp-Phe-Lys-Thr-Asp-Leu)**	**10**	**C52 H82 N12 O19**	**1178**	**30.22**
16	ALNQAWAFLK (Ala-Leu-Asn-Gln-Ala-Trp-Ala-Phe-Leu-Lys)	10	C56 H84 N14 O13	1160	57.47
17	AERVGAGAPVY (Ala-Glu-Arg-Val-Gly-Ala-Gly-Ala-Pro-Val-Try)	11	C48 H76 N14 O15	1088	35.06
18	RIVDFHMLESR (Arg-Ile-Val-Asp-Phe-His-Met-Leu-Glu-Ser-Arg)	11	C61 H99 N19 O17 S1	1401	41.94
19	SGVTIAQGGVLP (Ser-Gly-Val-Thr-Ile-Ala-Gln-Gly-Gly-Val-Leu-Pro)	12	C48 H83 N13 O16	1097	68.00
20	APRKQLATKAAR (Ala-Pro-Arg-Lys-Gln-Leu-Ala-Thr-Lys-Ala-Ala-Arg)	12	C56 H103 N21 O15	1309	94.82
21	QNIIPASTGAAK (Gln-Asn-Ile-Ile-Pro-Ala-Ser-Thr-Gly-Ala-Ala-Lys)	12	C50 H87 N15 O17	1169	44.40
22	HLQLAIRNDEELNK (His-Leu-Gln-Leu-Ala-Ile-Arg-Asn-Asp-Glu-Glu-Leu-Asn-Lys)	14	C72 H121 N23 O24	1691	83.11
23	VTIAQGGVLPNIQA (Val-Thr-Ile-Ala-Gln-Gly-Gly-Val-Leu-Phe-Asn-Ile-Gln-Ala)	14	C61 H105 N17 O19	1379	74.36

* EIA10, which has the lowest lipid accumulation, is shown in bold in the table.

**Table 2:** Effects on body weight, liver weight, adipose tissue, and biochemical parameters.

	CON	HFD	HFD+EIA10	HFD+Orlistat
Initial body weight (g)	22.61 ± 2.56	22.47 ± 0.36	23.07 ± 0.33	23.55 ± 0.07
Final body weight (g)	28.41 ± 0.15	43.12 ± 0.09 ^##^	37.38 ± 2.55 **	36.11 ± 3.92 **
Body weight gain (g)	5.80 ± 0.33	20.65 ± 0.92 ^##^	12.31 ± 2.54 **	12.56 ± 0.45 **
Total energy intake (kcal)	935.19 ± 43.12	1062.21 ± 16.19 ^##^	1026.01 ± 2.60	1031.35 ± 26.32
Epididymal adipose tissue weight (g)	0.67 ± 0.17	2.19 ± 0.28 ^##^	2.06 ± 0.34 **	1.90 ± 0.48 **
Subcutaneous adipose tissue weight (g)	0.66 ± 0.31	4.76 ± 0.39 ^##^	3.36 ± 0.58 **	3.11 ± 0.53 **
Interscapular adipose tissue weight (g)	0.19 ± 0.05	0.26 ± 0.04 ^#^	0.22 ± 0.06	0.26 ± 0.04 *
Total fat weight (g)	3.99 ± 0.71	18.15 ± 0.33 ^##^	12.71 ± 0.78 **	13.71 ± 1.09 **
Liver weight (g)	1.23 ± 0.27	1.72 ± 0.16 ^##^	1.22 ± 0.25 **	1.14 ± 0.12 **
TG (mg/dL)	109.44 ± 2.60	248.74 ± 4.62 ^##^	140.25 ± 5.23 **	115.52 ± 4.66 **
TC (mg/dL)	95.06 ± 2.69	132.65 ± 4.52 ^##^	107.46 ± 5.15 **	113.26 ± 3.40 **
HDL (mg/dL)	75.81 ± 2.64	52.35 ± 3.19 ^##^	74.49 ± 2.57 **	72.05 ± 4.38 **
LDL/VLDL (mg/dL)	19.25 ± 3.71	80.30 ± 4.12 ^##^	32.97 ± 3.42 **	41.21 ± 2.59 **
Glucose (mg/dL)	121.13 ± 8.41	175 ± 9.75 ^##^	137 ± 11.47 **	160.78 ± 13.62 **
ALT (U/L)	45.38 ± 8.10	98.50 ± 13.54 ^##^	58.75 ± 13.16 **	44.75 ± 8.38 **
AST (U/L)	110.00 ± 10.90	159.63 ± 9.26 ^##^	99.20 ± 20.00 **	113.88 ± 16.17 **

All data represent mean values ± standard error of the mean. Significant differences at ^#^ *p* < 0.05, ^##^ *p* < 0.05 compared with the control group. Significant differences at * *p* < 0.05, ** *p* < 0.01 compared with the HFD group. TG: triglyceride; TC: total cholesterol; HDL: high-density lipoprotein cholesterol; LDL: low-density lipoprotein cholesterol; VLDL: Very low-density lipoprotein cholesterol; ALT: alanine aminotransferase; AST: aspartate aminotransferase.

**Table 3:** Reaction conditions of different enzymes.

	Abbreviation	Temperature (°C)	pH	Addition (%)	Inactivation
Pancreatin	P	50	7	1	80 °C, 10 min
Food Pro Alkaline protease	AK	50	7	1	80 °C, 10 min
Alphalase NP	AP	50	7	1	80 °C, 10 min
Promod 278P	PM	50	7	1	80 °C, 10 min
Alcalase	AC	50	7	1	80 °C, 10 min
Neutrase	NT	50	7	1	80 °C, 10 min
Protamex	PMX	50	7	1	80 °C, 10 min
Protease NP	PNP	50	7	1	80 °C, 10 min
Trypsin	TS	38	7	1	80 °C, 10 min
Pepsin	PS	38	2 (HCl 35%)	1	pH 7.5 (NaOH 5N)
Control	-	50	7	-	80 °C, 10 min

**Table 4:** Primers used in reverse transcriptase–polymerase chain-reaction analysis.

Gene Name		Sequence
*C/EBP-α*	Forward	5-CGT CTA AGA TGA GGG AGT C-3
	Reverse	5-GGC ACA AGG TTA CTT CCT-3
*PPAR-γ*	Forward	5-GAA AGA CAA CGG ACA AAT CAC-3
	Reverse	5-GAA ACT GGC ACC CTT GAA-3
*FAS*	Forward	5-CTT GGG TGC TGA CTA CAA CC-3
	Reverse	5-GCC CTC CCG TAC ACT CAC TC-3
*SREBP-1c*	Forward	5-CTT CTG GAG ACA TCG CAA AC-3
	Reverse	5-GGT AGA CAA CAG CCG CAT C-3
*GAPDH*	Forward	5-GCA CAG TCA AGG CCG AGA AT-3
	Reverse	5-GCC TTC TCC ATG GTG GTG AA-3

*C/EBP-α*: CCAAT/enhancer-binding protein-*α*; *PPAR-γ*: peroxisome proliferator-activated receptor-γ; *FAS:* fatty acid synthase; *SREBP-1c:* Sterol regulatory element-binding protein 1; *GAPDH*: glyceraldehyde-3-phosphate dehydrogenase.

## Supplementary Materials

The following are available online at https://www.mdpi.com/1422-0067/21/22/8537/s1.

## Abbreviations

EIA10	Glu-Ile-Ala-Gln-Asp-Phe-Lys-Thr-Asp-Leu
FAO	Food and Agriculture Organization of the United Nations
TG	Triglyceride
EPHs	Enzymatic protein hydrolysates
Q-TOFMS	Quadrupole time-of-flight mass spectrometry
NEFAs	Non-esterified fatty acids
AUC	Area under the glucose–tolerance curve
*FAS*	Fatty acid synthase
*PPARγ*	Peroxisomal proliferator-activated receptor γ
PNP	Protease NP
P	Pancreatin
AP	Alphalase NP
AK	Alkaline protease
PM	Promod 278P
AC	Alcalase
NT	Neutrase
PMX	Protamex
FPLC	Fast protein liquid chromatography
DMEM	Dulbecco’s modified Eagle medium
P/S	Penicillin/streptomycin
IEC	Ion-exchange chromatography
ACN	Acetonitrile
Fmoc	Fluorenylmethylocycarbonyl
tBu	tertiary-butyl
HPLC	High-performance liquid chromatography
LC	Liquid chromatography
MS	Mass spectrometry
HFD	High-fat diet
DXA	Dual-energy X-ray absorptiometry
H&E	Hematoxylin and eosin
ALT	Alanine aminotransferase
AST	Aspartate aminotransferase
LDL	Low-density lipoprotein cholesterol
HDL	High-density lipoprotein cholesterol
ELISA	Enzyme-linked immunosorbent assay
OGTT	Oral glucose tolerance test
Hepatic IRI	Hepatic insulin resistance index
HOMA-IR	Homeostasis model assessment of insulin resistance
RT	Reverse transcription
PCR	Polymerase chain reaction
cDNA	Complementary DNA
*GAPDH*	Glyceraldehyde 3-phosphate dehydrogenase
ANOVA	Analysis of variance
