# Supplementary material for: Exploring the Role of a Novel Peptide from Allomyrina dichotoma Larvae in Ameliorating Lipid Metabolism in Obesity
Source: Int J Mol Sci. 2020 Nov 12;21(22):8537. doi: 10.3390/ijms21228537 (PMC7698306; doi:10.3390/ijms21228537)
Supplement: Supplementary file 1 [file ijms-21-08537-s001.zip › ijms-972980-supplementary.pptx]

## Slide 1
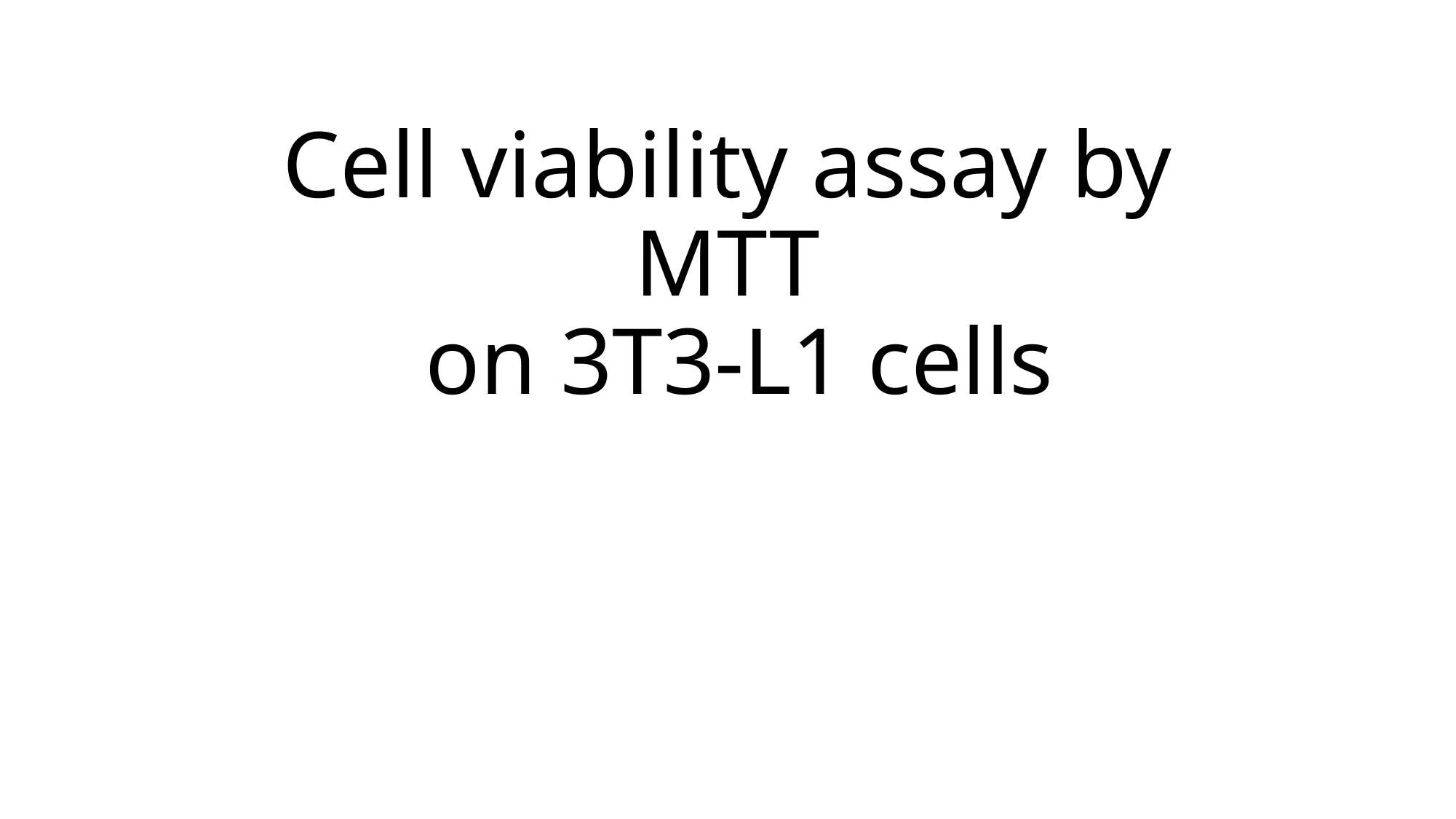

# Cell viability assay by MTT on 3T3-L1 cells

## Slide 2
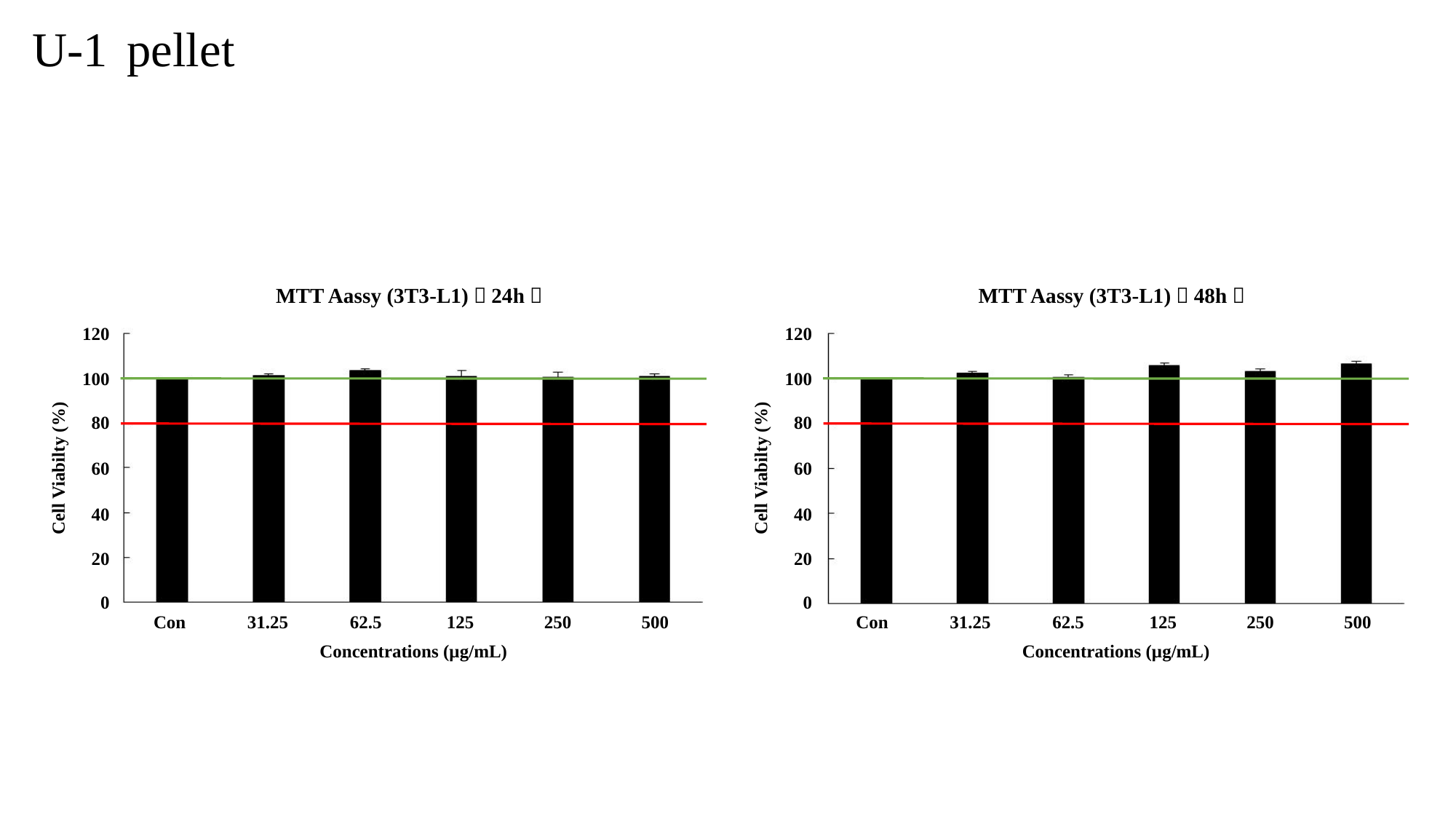

U-1 pellet
MTT Aassy (3T3-L1)（24h）
Cell Viabilty (%)
120
100
80
60
40
20
0
Con
31.25
62.5
125
250
500
Concentrations (μg/mL)
MTT Aassy (3T3-L1)（48h）
Cell Viabilty (%)
120
100
80
60
40
20
0
Con
31.25
62.5
125
250
500
Concentrations (μg/mL)

## Slide 3
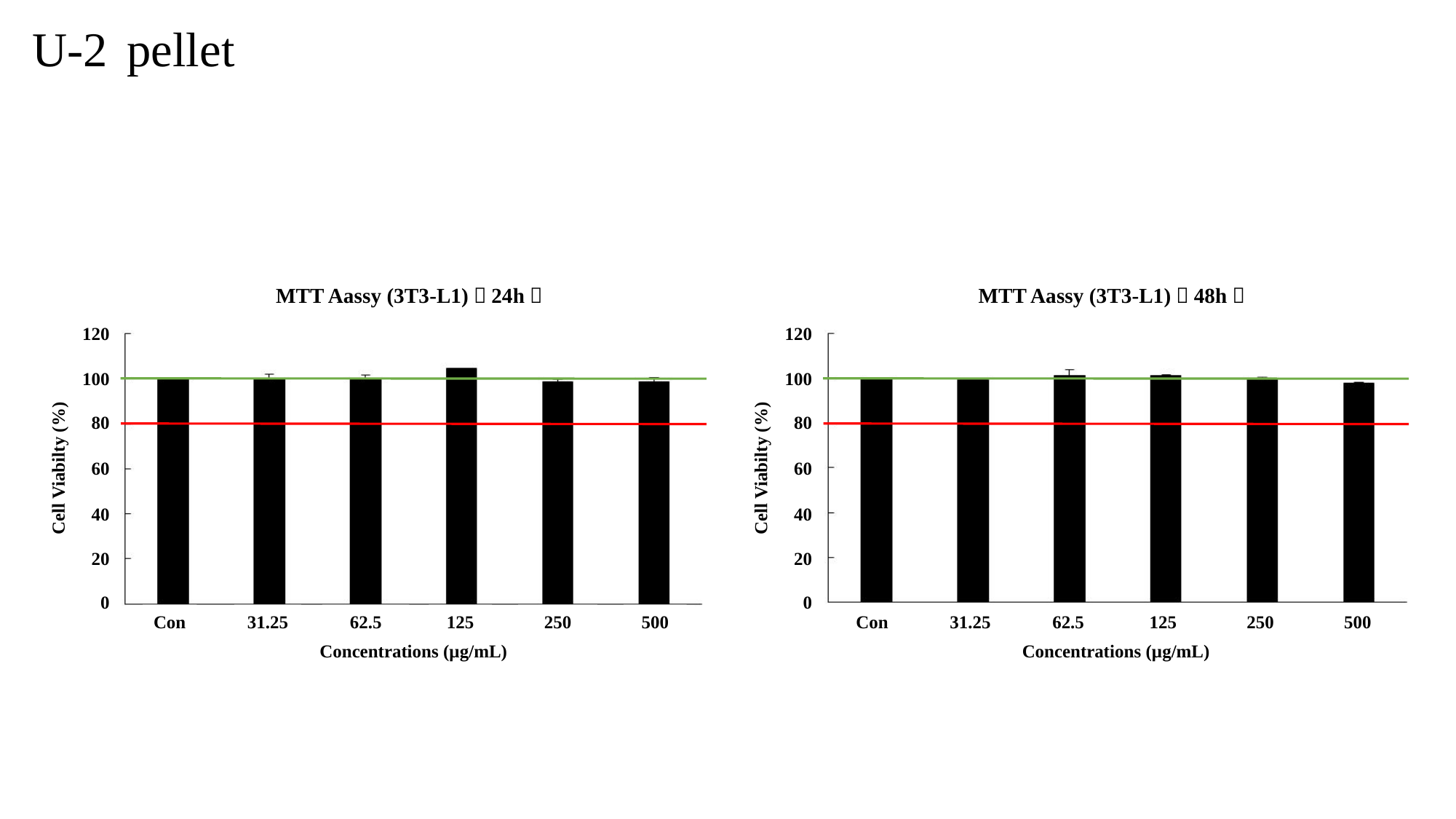

U-2 pellet
MTT Aassy (3T3-L1)（24h）
Cell Viabilty (%)
120
100
80
60
40
20
0
Con
31.25
62.5
125
250
500
Concentrations (μg/mL)
MTT Aassy (3T3-L1)（48h）
Cell Viabilty (%)
120
100
80
60
40
20
0
Con
31.25
62.5
125
250
500
Concentrations (μg/mL)

## Slide 4
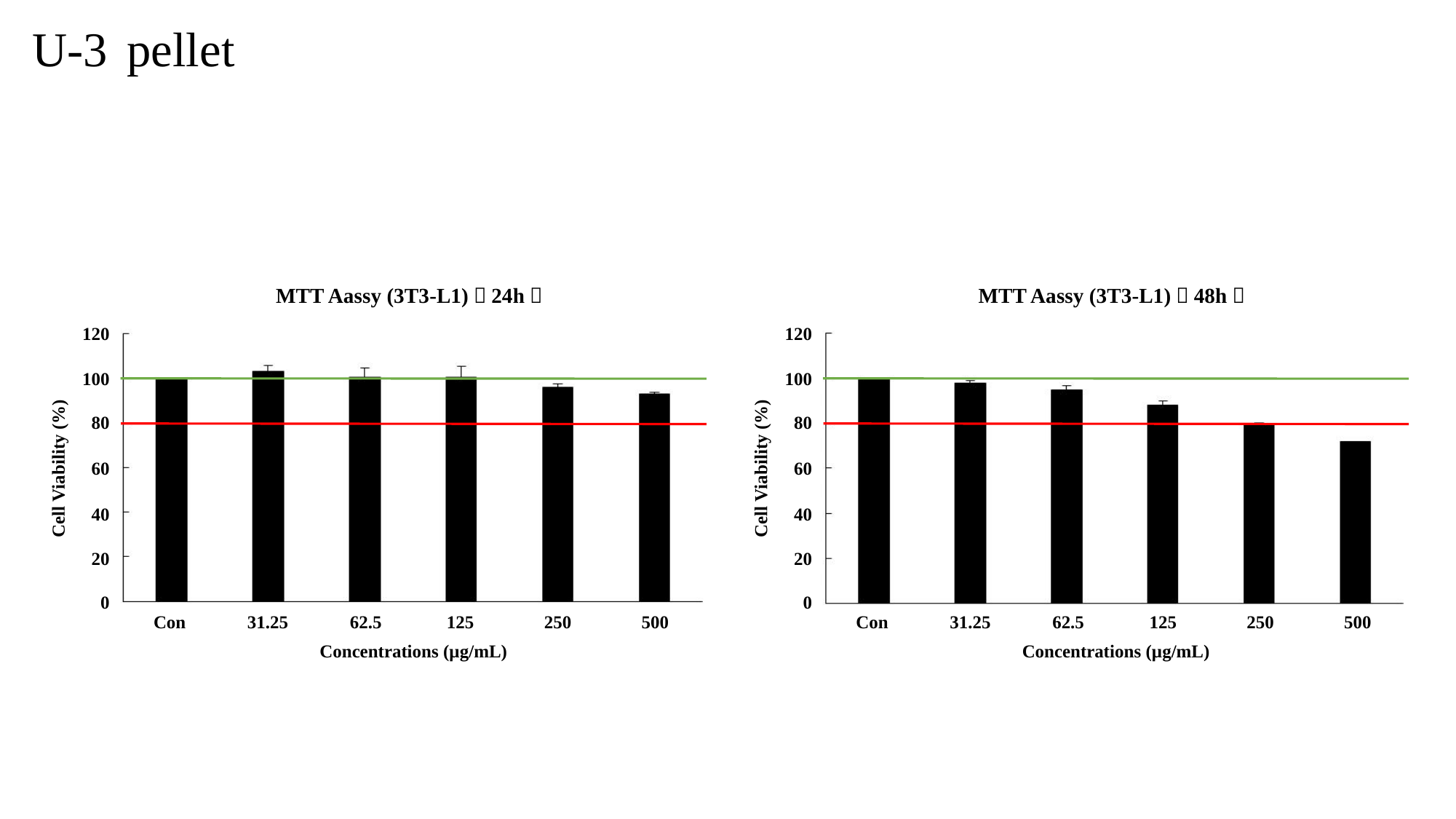

U-3 pellet
MTT Aassy (3T3-L1)（24h）
Cell Viability (%)
120
100
80
60
40
20
0
Con
31.25
62.5
125
250
500
Concentrations (μg/mL)
MTT Aassy (3T3-L1)（48h）
Cell Viability (%)
120
100
80
60
40
20
0
Con
31.25
62.5
125
250
500
Concentrations (μg/mL)

## Slide 5
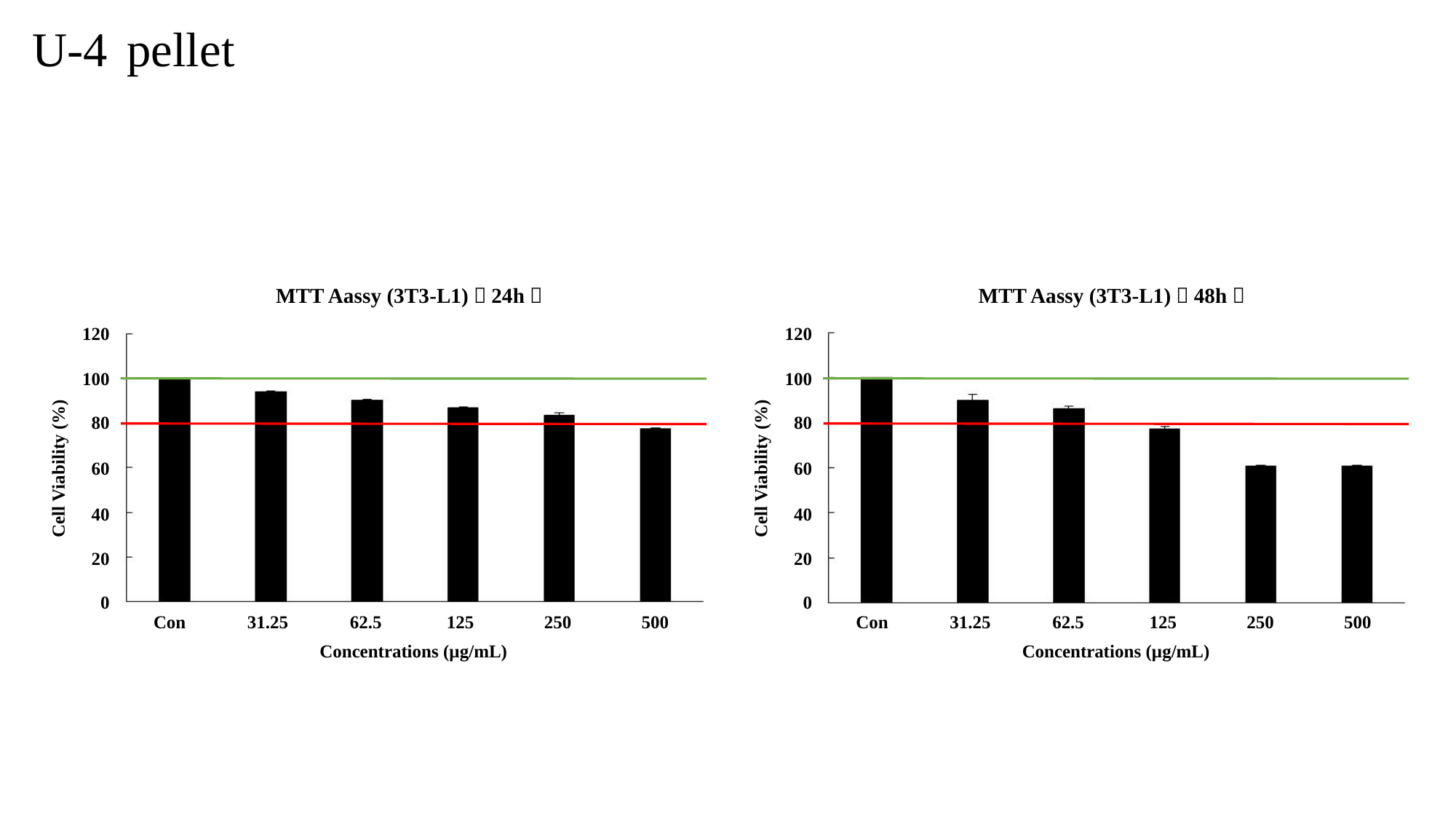

U-4 pellet
MTT Aassy (3T3-L1)（24h）
Cell Viability (%)
120
100
80
60
40
20
0
Con
31.25
62.5
125
250
500
Concentrations (μg/mL)
MTT Aassy (3T3-L1)（48h）
Cell Viability (%)
120
100
80
60
40
20
0
Con
31.25
62.5
125
250
500
Concentrations (μg/mL)

## Slide 6
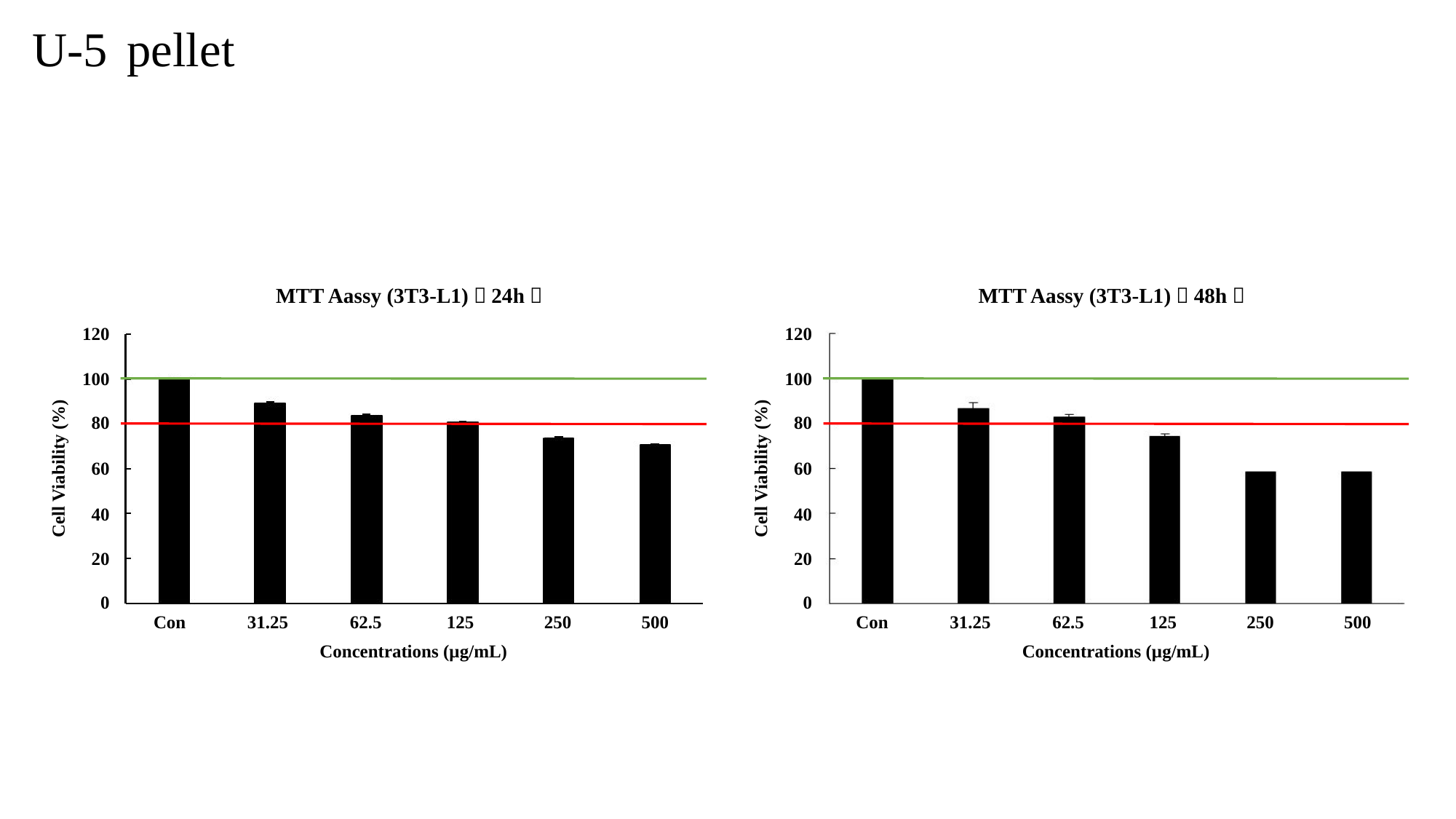

U-5 pellet
MTT Aassy (3T3-L1)（24h）
Cell Viability (%)
120
100
80
60
40
20
0
Con
31.25
62.5
125
250
500
Concentrations (μg/mL)
MTT Aassy (3T3-L1)（48h）
Cell Viability (%)
120
100
80
60
40
20
0
Con
31.25
62.5
125
250
500
Concentrations (μg/mL)

## Slide 7
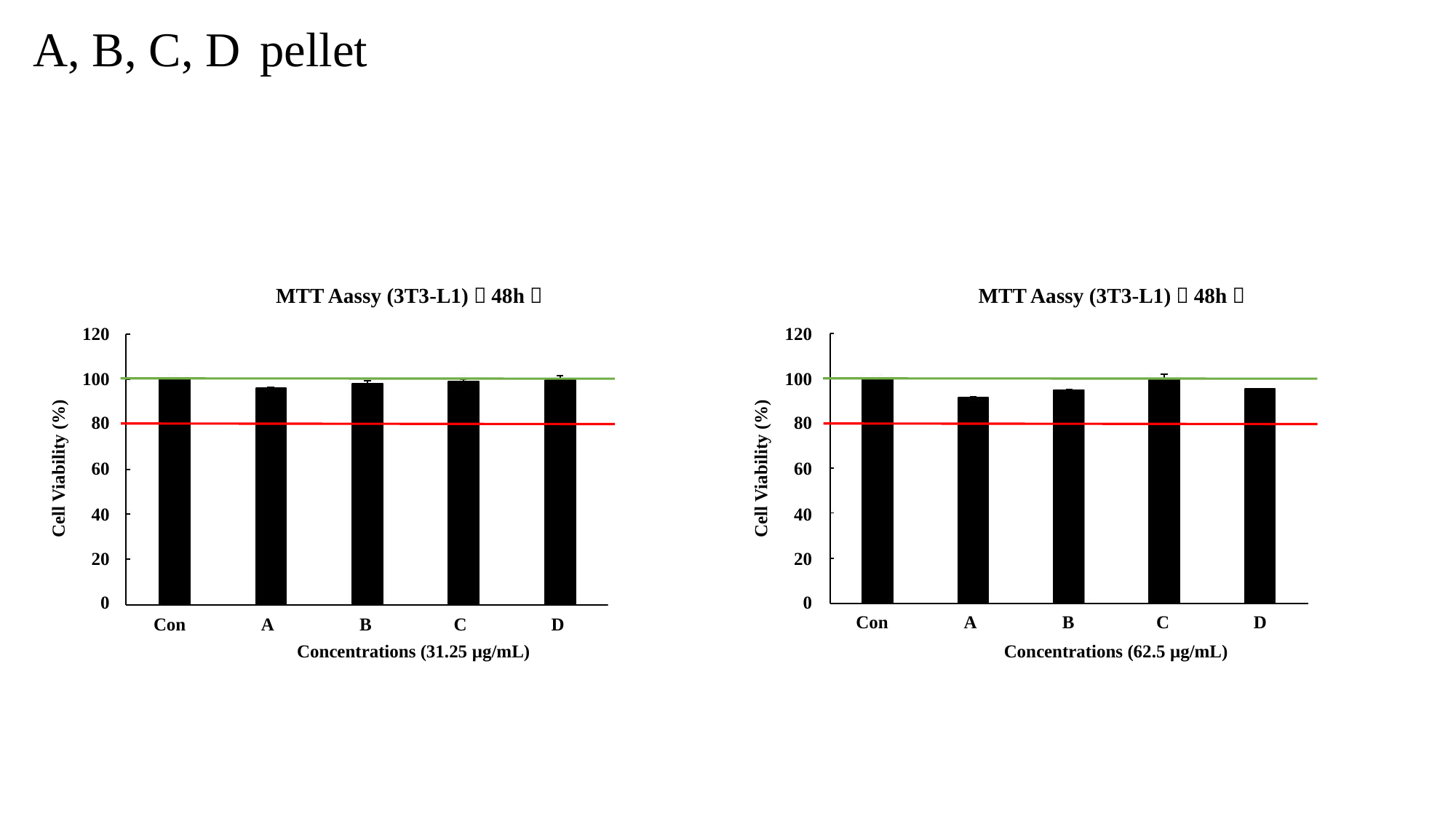

A, B, C, D pellet
MTT Aassy (3T3-L1)（48h）
Cell Viability (%)
120
100
80
60
40
20
0
Con
A
B
C
D
Concentrations (31.25 μg/mL)
MTT Aassy (3T3-L1)（48h）
Cell Viability (%)
120
100
80
60
40
20
0
Con
A
B
C
D
Concentrations (62.5 μg/mL)

## Slide 8
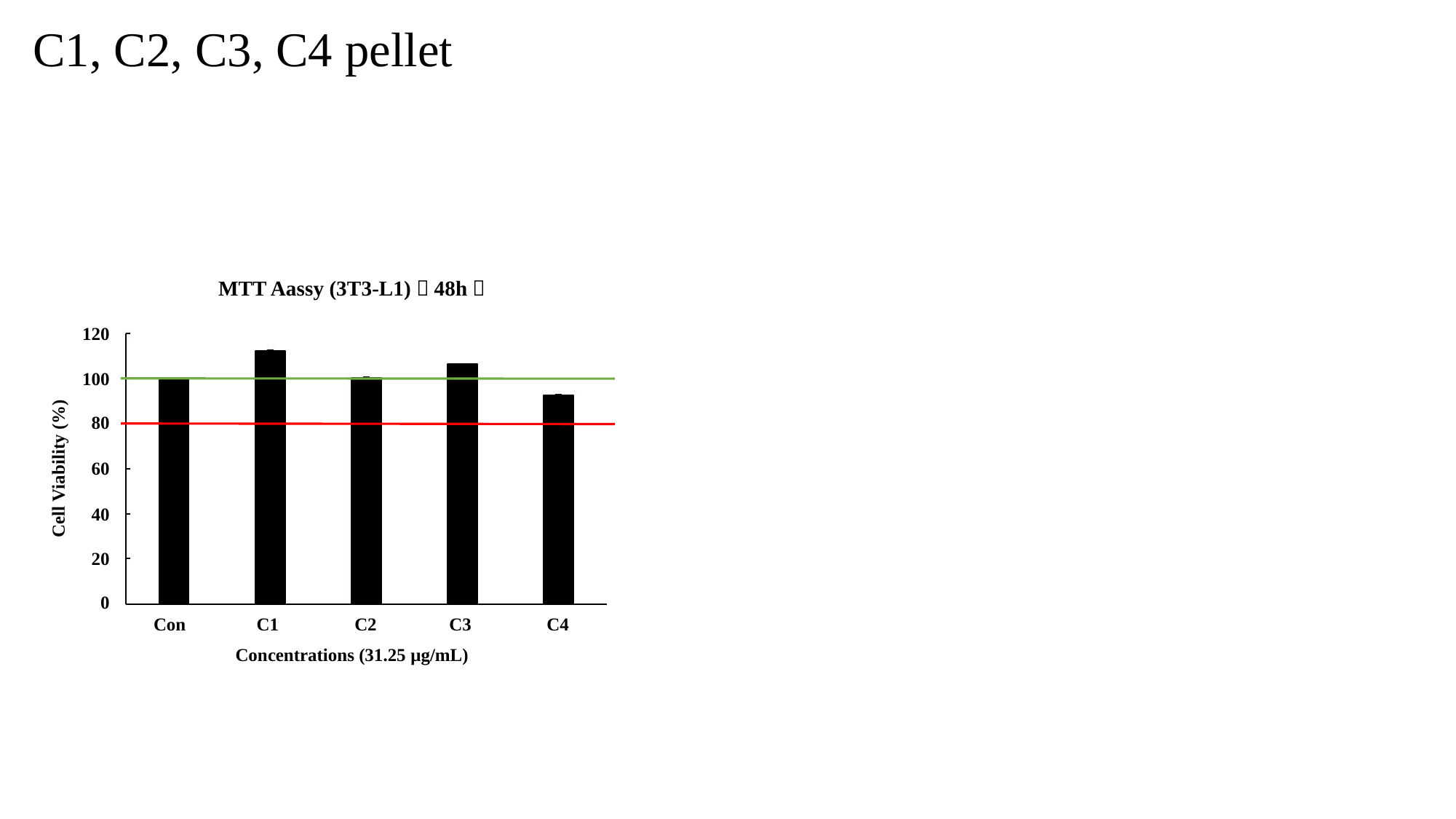

C1, C2, C3, C4 pellet
MTT Aassy (3T3-L1)（48h）
Cell Viability (%)
120
100
80
60
40
20
0
Con
C1
C2
C3
C4
Concentrations (31.25 μg/mL)

## Slide 9
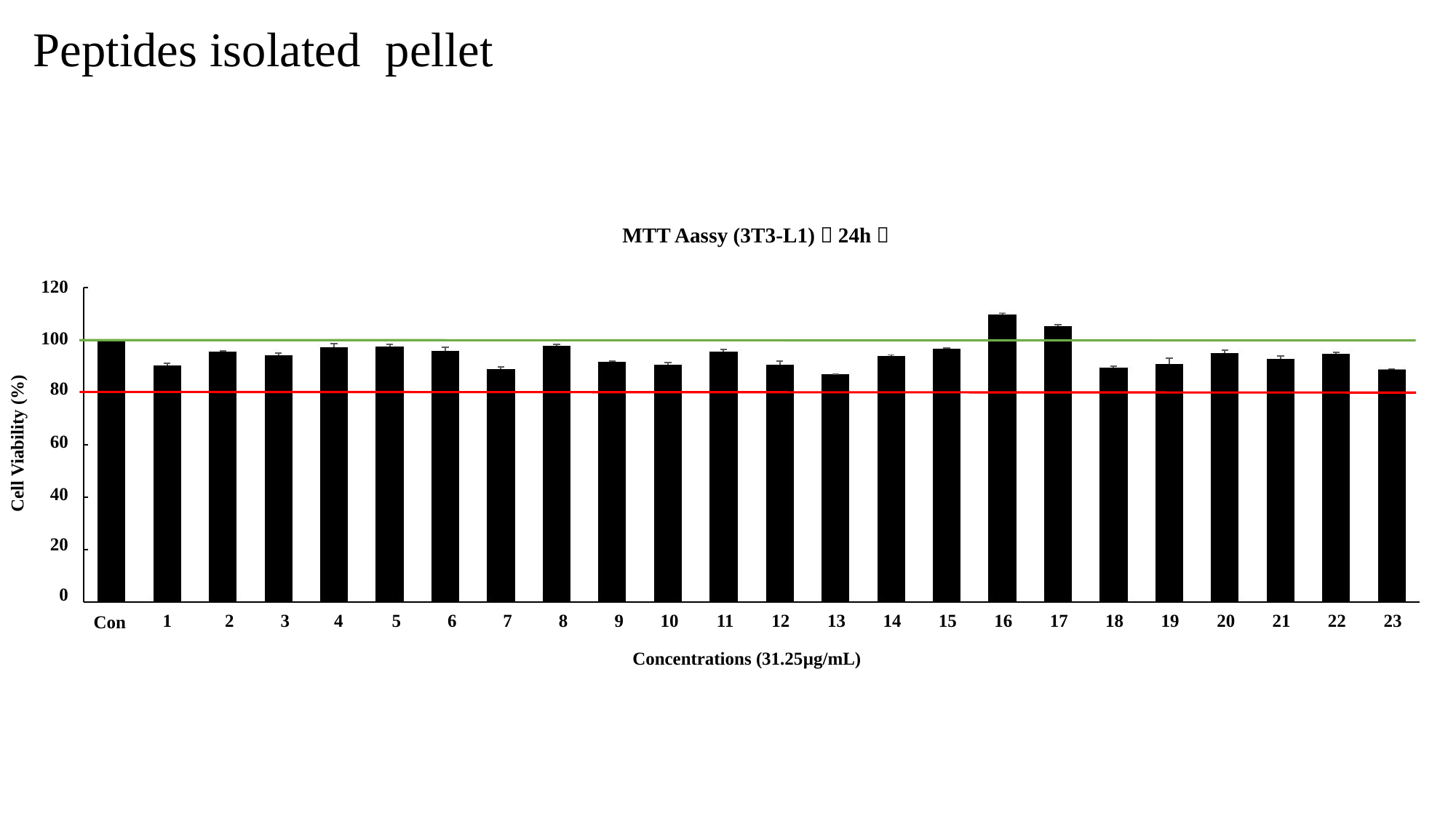

Peptides isolated pellet
MTT Aassy (3T3-L1)（24h）
Cell Viability (%)
120
100
80
60
40
20
0
1
2
3
4
5
6
7
8
9
10
11
12
13
14
15
16
17
18
19
20
21
22
23
Con
Concentrations (31.25μg/mL)

## Slide 10
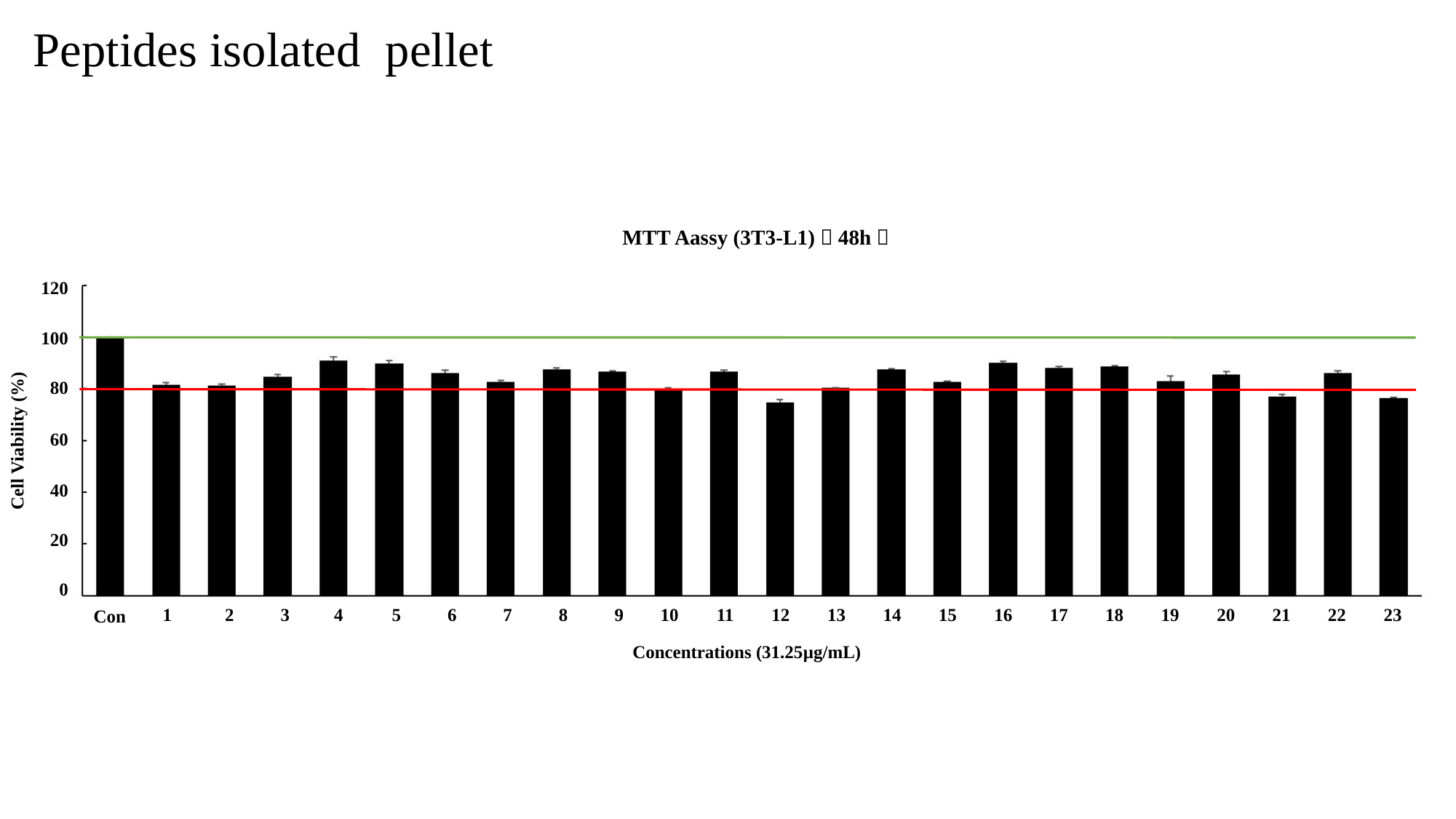

Peptides isolated pellet
MTT Aassy (3T3-L1)（48h）
Cell Viability (%)
120
100
80
60
40
20
0
1
2
3
4
5
6
7
8
9
10
11
12
13
14
15
16
17
18
19
20
21
22
23
Con
Concentrations (31.25μg/mL)
